# Observations on Italian Medical Institutions and Practice; a Brief Comparative Sketch of Some Points of French and English Practice, and Remarks on Italian Climate

**Published:** 1831-10

**Authors:** Edwin Lee

**Affiliations:** Member of the Royal College of Surgeons, and formerly House Surgeon to St. George's Hospital.


### ITALIAN MEDICAL INSTITUTIONS AND PRACTICE.

*Observations on Italian Medical Institutions and Practice; a brief Comparative Sketch of some Points of French and English Practice, and Remarks on Italian Climate.* By Edwin Lee, Esq., Member of the Royal College of Surgeons, and formerly House Surgeon to St. George's Hospital.

I purpose, in the present paper, giving an account of some of the Italian hospitals and medical institutions, and attempting to convey an idea, by the observations which I made during my residence in Italy, and by the relation of some cases selected from hospital practice, of the actual state of medicine and surgery in that country. I shall also offer a few remarks on some of the more prominent differences in medicine and surgery, observable between this country and France: the French medical institutions, and the practice of individual professors, it is not my intention to describe, as two or three publications already exist on that subject.* I also add a few remarks on the climate of particular parts of Italy.

Although aware of the incompleteness of the following notes, which were taken as an occupation of leisure hours, without any intention of publication, yet, as no account of the Italian medical establishments has been published in England, (with the exception of some observations in Dr. Clarke's "Notes,") these observations, however imperfect, may perhaps, in the absence of other information, be not altogether void of interest.

I beg here to express my grateful sense of the kindness and urbanity I experienced from the professors and medical attendants of the different hospitals which I visited; and assure future travellers, that, on the continent generally, no impediment is offered to their visiting charitable institutions, but, on the contrary, every facility of investigation will be afforded them.

### *Brief Sketch of some Points of French and English Practice.*

Almost all the hospitals and medical institutions on the continent are supported, either directly or indirectly, by the government of their respective countries, and patients obtain admission or relief on application, without any other recommendation than that of their requiring professional assistance. In England, where establishments for the relief of the sick poor partake more of the nature of private institutions, a letter of recommendation from one of the subscribers is requisite for the admission of patients, except in cases of accident. No circumstance can more strongly portray the benevolent disposition of the English nation than the existence of the numerous charitable institutions in all parts of the kingdom, which are, with very few exceptions, supported entirely by the subscriptions and donations of individuals.

On the continent, the medical officers of hospitals are elected by "concours," or the public examination of candidates: this method has certainly many advantages; it affords a guarantee to hospital patients of the skill of their professional attendants; and, as medical institutions are strictly public, no other plan could be so well followed. In England, however, where these establishments are supported by the benevolence of individuals, who are naturally entitled to take part in their internal management, the election of candidates

* Cross's Sketches on Paris; Ratier, Coup d'Œil sur les Hôpitaux de Paris.

is decided by the majority of the votes of the subscribers; and I question whether the voice of public opinion and criticism, far more strongly expressed in England than elsewhere, to which medical officers of charitable institutions are exposed, and the judicial investigation which must ensue on every fatal case, where a doubt exists as to the propriety of treatment, be not securities of the capabilities of hospital medical officers, quite as valid as those afforded by the election by "concours;" and whether they would not, more certainly, deter incompetent persons from proposing themselves as candidates.

The profession does not hold so high a rank in society on the continent as in England: it does not frequently happen abroad, that any one possessing much property selects the medical profession, while the trifling expense of professional education allows admission to many of the lower class of individuals, who, in England, would be excluded by the heavy expenses attendant on their education. The bodies of all patients who die in the continental hospitals are examined; hence immense opportunities are afforded for the cultivation of pathological anatomy, of which the French have most zealously availed themselves; without, however, a corresponding amelioration in the treatment of disease taking place, to the extent which might have been expected.

From the same source, the dissecting rooms are plentifully supplied; and it may be questioned if this abundance of subjects have not its disadvantages, from the inducement it offers to students to hurry through their dissection in a careless manner: a principal cause of the greater neatness in dissection displayed in the London schools, is no doubt owing to the scarcity of subjects.

The majority of the profession in France follow in the treatment of disease the doctrine of Broussais; of which the leading feature is the reference of fever to a local origin. This is considered to be inflammation, in a greater or less degree, or irritation of the mucous membrane of the stomach and bowels, which is supposed to be present in the greater number of acute, and in many chronic diseases: hence the practice mainly consists in the avoidance of all kinds of stimulants and tonics, of active purgation, or of any medicine which would be likely to irritate this membrane; and in the employment of light farinaceous or liquid diet, occasional bloodletting, the repeated application of leeches to the abdomen, and the administration of acidulated or mucilaginous drinks.

A smaller proportion of practitioners of the old school of

Pinel continue to follow the system of the "medicine expectante," or "Hippocratique," rarely using depletory measures, relying chiefly on the "vis medicatrix naturæ," and, even in cases of acute inflammation, confining their remedial efforts to a small bleeding, the application of leeches, the exhibition of an emetic, or the use of revulsives. Tisanes, decoctions of simple herbs, mucilaginous beverages, are the most usual remedies employed; joined to the use of enemata, tepid baths, and, in those cases where debility is present, of tonics and stimulants.

Many judicious practitioners, however, do not follow the extremes of any system, but, on prescribing according to existing symptoms and the circumstances of each particular case, endeavour to attain the "juste milieu" in the treament of disease: in fact, the too exclusive nature of the Broussaian doctrine has of late years somewhat lessened the number of its followers.

One material difference in the treatment of disease, between England and the continent, consists in the much larger quantity of medicine of all kinds, and particularly of mercurial preparations, prescribed for patients in this country; a practice which may owe its origin to the manner of renumerating the majority of practitioners, (viz. in proportion to the quantity of medicine sent): and to the notion which is thus entertained of the necessity of quantities of medicine on every slight occasion, may doubtless be traced the habit which the English have acquired of doseing themselves with active remedies, on the most trivial deviation from a state of health.

Although in England the use of medicines is on some occasions carried to an injurious extent, yet, in acute disease, continental practitioners might perhaps find their advantage in adopting the English practice of using more freely those remedies which have the effect of increasing the secretions. This might have been inferred from the benefits derived from the exhibition of large doses of antimony, as used in Italy, which was made to supersede active depletion in many cases of acute inflammation. In France and Italy, antimonials are not unfrequently employed; calomel and active purgatives very rarely; blue pill scarcely ever heard of; and the class of antispasmodics, stimulants, and tonics, of such signal benefit in many nervous, dyspeptic, and other cases, is almost completely proscribed by the advocates of the Broussaian doctrine.

Bloodletting is a remedy very differently employed in England, to what it is on the continent. In this country, if a strong person be attacked by acute inflammation of an important organ, a full bleeding, from fourteen to twenty ounces, generally succeeds in checking the progress of the disease, and in many cases the necessity of its repetition is obviated; in those cases where the inflammation is not subdued, the bleeding is repeated, the quantity of blood abstracted being proportioned to the violence of the disease and strength of the patient; this, together with the use of purgatives, antimonials, &c., procures the speedy recovery of the patient in an immense majority of cases.

On the continent, when bloodletting is employed, the quantity of blood abstracted at the outset very rarely exceeds ten ounces: this, in a large proportion of cases, is not sufficient to arrest effectually the progress of the inflammation; hence the more frequent necessity of further depletion, which is effected either by the abstraction of a smaller quantity of blood from the system, or more commonly by the repeated application of leeches; the patient at the same time taking an acidulated beverage or a decoction of simple herbs, with, perhaps, an occasional dose of castor oil; so that although the continental patient may lose as much or more blood in the aggregate, than the English patient, it is less calculated to produce the desired effect; while the neglect of the means of producing increased secretion from the bowels necessitates the more frequent abstraction of blood, and the patient, in the favorable cases, has to support a tedious convalescence.

Another striking difference is in the treatment of continued fever. The disciples of Broussais see nothing but gastroenterite as the cause of the general disturbance of the functions, and continue the application of leeches and the use of acidulated or emollient drinks, even when the fever has assumed the typhoid type. How can they reconcile the existence of inflammation of the stomach, with the benefit which is derived from active purgation in many cases, and with the recoveries which so frequently take place in the advanced stage of the disease, from the employment of tonic and stimulant remedies.

Intermittents are considered by the followers of Broussais to depend on periodical irritation of the mucous membrane of the stomach and bowels; yet there is no more evidence of increased irritation of this membrane at the period of the paroxysm, than during the intermissions of the fever; nor can this theory be reconciled with the fact of bark being a specific in intermittents, and as such it is used almost as universally on the continent as in England.

There exist also material differences between England and France in several points connected with surgical disease.

One of the principal is the treatment of many local diseases by constitutional means, employed by English practitioners; such as the medical treatment of chronic inflammation in various textures, cachectic diseases and ulcers, some tumors of the breast, chronic disease of the testicle, many nervous complaints, periosteal inflammation, diseases of the eyes; constitutional irritation, either supervening on an operation or injury, or occurring during the progress of disease, as stone in the bladder, &c.

In France and Italy, scarcely any medicine is given in the majority of surgical complaints; the means of relief being generally restricted to rest, the abstraction of blood, local applications, or operation.

Less attention is also paid to the establishment of a correct diagnosis in diseases of the joints than in England: in France the generic appellation of "tumeurs blanches" is almost universally applied to these complaints: the treatment usually consists in rest of the part, the local abstraction of blood, emollient applications, blistering, and, in the more chronic forms of disease, the use of the moxa.

M. Roux, in his "Parallèle," accuses the English of not paying sufficient attention to the preparation of patients by diet, medicine, &c., previous to the performance of operations. However this may have been at the period of his visit, at the present time English surgeons cannot be justly accused of underrating the importance of preparatory measures, and I am inclined to think that more care is taken in England than on the continent to ascertain, previous to the performance of an operation, that the patient be in a fit state, with respect to his general health, and freedom from constitutional or organic disease, to undergo the operation with advantage.

The practice of treating wounds, whether from accident or operation, on the principle of union by the first intention, is not frequently followed in France by the majority of surgeons.

M. Roux attributes the universal adoption of this practice in England, to our not having a sufficiency of the materials necessary for the daily dressing of large wounds in a state of suppuration; although he acknowledges its advantages in many instances,—that the danger and attendant inflammation are much lessened; that the wound more quickly heals when this practice is pursued; and that, even in those cases where union does not take place, the wound is much diminished, the suppuration less copious, the local and general irritation less severe, than in those cases where the wound is healed by the suppurative process. Nevertheless, he accuses us of applying the practice too indiscriminately; but does not support his statement by any valid arguments, nor point out any serious ill consequences from the failure of the attempt. Every one will agree with him, that immediate union is counter-indicated in many wounds, and few would attempt it in gunshot wounds, in deep punctured wounds, or in extensive lacerated wounds attended with loss of substance; but the objections he makes to its being attempted after the operation of castration, or the relation of a particular case which he saw in England, in which sutures were used, and union did not take place, in consequence of an attack of inflammation of the part, will not deter many who have had opportunities of judging of the merits of the practice employed in both countries, from attempting union, which, in the great majority of cases, takes place partially after this operation, is attended with trifling constitutional disturbance; and, even in the few cases where it fails altogether, leaves the patient in no worse condition than those with whom the practice of filling the scrotum with charpie (which is not unfrequently attended with a dangerous degree of irritation,) is followed. The occurrence of subsequent hemorrhage might indeed be a good reason for not attemping union after this operation, were not care taken to prevent its happening, by placing a fine ligature on each of the small bleeding vessels of the scrotum.

The union of the wound after the ligature of a vessel for aneurism, is considered by M. Roux as another abuse of the practice: he says, "if such a wound be united with the view of obtaining a speedy cure, it will be necessary to apply no more ligatures than what are absolutely necessary to intercept the passage of the blood; the application of supernumerary ligatures for the prevention of hemorrhage, should it afterwards come on, is incompatible with the immediate union of the wound; at least it would be in the highest degree ridiculous to combine these two things; but to renounce the supernumerary ligatures for the purpose of remedying consecutive hemorrhage, is to deprive ourselves of the most simple remedy in case such an accident should occur; it is sacrificing an important resource to the poor advantage of a speedy cure; it is compromising the success of the operation."*

It would appear from the above passage, that, at the time of M. Roux's visit to England, a principle on which arteries are tied in this country, viz. that of detaching the artery as little as possible from its surrounding connexions, discarding all ligatures of reserve, and thereby greatly lessening the chances of consecutive hemorrhage, was not known in France:

* Parallèle de Chirurgie, &c.

in fact, the principles which guide our operations on arteries are far from being generally understood on the continent at the present day. Secondary hemorrhage is of much rarer occurrence after operations for aneurism, ever since a single round ligature has been used, and in those cases where it has occurred, the cause could generally be traced to a diseased state of the coats of the vessel, to the artery having been too much separated from its connexions, and consequently deprived of the supply of blood necessary for its support; or to the ligature having been placed immediately below the origin of a large branch, thereby preventing the formation of a sufficiently firm coagulum within the vessel.

M. Roux acknowledges the advantages of the English practice in the treatment of ulcers of the legs by adhesive plaster and bandage, and has adopted it at La Charité; yet this method is not applicable in all cases. Some ulcers of the legs are cured here by the means generally employed on the continent, viz. rest, emollient cataplasms, or simple dressings; and others by medicines tending to improve the general health, with stimulating applications to the part: this merely proves, however, that the cases require discrimination in the adaptation of the remedies.

Fractures of the bones are at the present day treated pretty much alike in both countries. Almost all the French sur geons maintain the possibility of union taking place in fracture of the neck of the thighbone within the capsular ligament. M. Roux censures the practice in fractures of the lower extremity, more generally followed formerly than at present, of placing the limb in a state of semiflexion, and resting on its outer side; and he infers, from having seen in London two cases of false joints in consequence of ununited fracture, the frequent occurrence of these accidents in England. It is seldom that we meet with, at present, even a false articulation of the humerus, where non-union of fractures occurs more readily than in other bones; most likely from this being the only fracture in which the weight of the limb tends to separate the extremities of the bone, unless care be taken to counteract this cause, by giving due support to the elbow.

An improvement applicable to simple fractures of the leg has lately been adopted in several cases in London: I mean the apparatus of Mr. Amesbury, in which, by means of the weight of the upper part of the limb being transferred to the ham, the patient is enabled to get about on crutches during the progress of the cure. In those cases which I have seen, consolidation appeared to be more quickly effected than in the treatment by the ordinary method.

Baron Larrey treats compound fractures of the leg in the following manner, and, as he states, with very successful results; although, from its not being more generally adopted, it may be inferred that the practice is not so successful in the hands of others. A junk is made with a piece of strong linen, and of pieces of straw bound firmly together, as in the London hospitals; on this are placed transversely three broad pieces of bandage; the limb is then placed on the junk, the foot resting on the heel; the Baron then covers the wound with a piece of rag, with holes cut in it, (*linge perforé,*) to allow the escape of any matter, which is absorbed by a quantity of charpie placed over the rag; a compress, wetted with a lotion composed of the white of three eggs, Oi. camphorated spirit, and Oij. Lotionis Plumbi, is then put over the whole; the transverse bandage, wetted with the lotion, is bound firmly round the limb, the straw splints are approximated and tied with tape, as in a common junk; the limb is then left to itself, and the apparatus never removed or opened, on any account, till the expiration of sixty days, when the cure will be found to be effected.

The mode of performing the lateral operation for the stone does not materially vary from that employed in this country, except that the knife, or bistouri caché, are more frequently used than the gorget. M. Dupuytren employs a bistouri caché with two blades, which divides the neck of the bladder and prostate gland on either side of the urethra. I believe this plan has but few followers: two cases in which I saw M. Dupuytren operate in this manner, under apparently favorable circumstances, terminated fatally, from the consequences of extravasation of urine about the neck of the bladder.

The operation for hernia is performed much earlier after strangulation in France than in this country, where, in general, the attempts to reduce the hernia by the taxis are too frequently repeated, and much time is lost in the trial of various remedies in succession: although these means succeed in many cases in effecting reduction, yet, when they fail, the chances of success from the operation are much diminished by the frequent handling of the tumor, and by the delay these efforts have occasioned; and it is better that an operation, not in itself dangerous, should be performed in some cases where it might be obviated, than that it should be employed as a "dernier resource," and delayed until the probabilities of recovery are greatly lessened.

In consequence of the early performance of the operation in France, the subsequent inflammation does not generally run so high as in this country: when, however, violent inflammation supervenes, the after-treatment pursued is less calculated to subdue it than the energetic practice adopted in England, by copious depletion and the use of purgatives.

Continental surgeons agree with the majority of English surgeons, in considering Gimbernat's ligament to be the chief cause of the constriction in the greater number of cases of femoral hernia. Sir A. Cooper, however, states that this ligament can never occasion the constriction, which is situated at the crural arch. But do not these parts form an *ensemble,* the division of one of which would relieve the state of tension in which they mutually support each other? Granting the seat of stricture to be in the femoral ring, does not the sharp unyielding ligament of Gimbernat form a material part of that ring, and would not its division tend to relax Poupart's ligament more than a partial division of this latter would relax Gimbernat's ligament? Doubtless in some cases the constriction is situated in the opening in the fascia lata for the passage of the vena saphena; but whether femoral hernia passes through this aperture in all cases, is a point which may be questioned.

Cold applications are not frequently used to reduce the temperature of parts in a state of inflammation, by French and Italian surgeons: this is the more surprising, as the beneficial effects of this remedy must be apparent on trial. The agency of heat is, however, frequently made use of, even in some cases where cold lotions would be applied in England: thus Baron Larrey uses the actual cautery in most cases of erysipelas, and the moxa is applied in some diseases of the joints, which are here benefited by cold applications.

Attempts have been made in some Parisian hospitals to cure scirrhous tumors by the use of compression. M. Recamier,* who has published the results of his experiments, even employs this method, joined to the cauterization or excision of cancerous vegetations, in open cancer. The results of this treatment have not, however, led to its adoption: in fact, in France, some tumors are considered to be cancerous, which would not be regarded as such by English surgeons; and it may be questioned if the majority of those cases in which material benefit was derived from this treatment, would not have been considered as simple chronic tumor in England. During my stay in Paris, I had an opportunity of seeing, at the hospital of La Pitié, a case of

* Recherches sur le Traitement de Cancer par la Compression; two vols. 1829. A brief analysis of this work is given in our last number.—Editor.

tumor of the breast, which was considered cancerous, and was certainly benefited by the use of compression; but the diagnostic symptoms of scirrhus were absent, and the patient was under thirty years of age.

An important invention has been brought forward by the French, that of lithotrity, or perforating and breaking a calculus within the bladder, which will have the effect of superseding the operation of lithotomy in many cases, particularly in young and middle-aged persons: in children it is not so admissible; in them the frequent introduction of instruments, and the necessary dilatation of the urethra, would occasion more irritation than the operation of lithotomy. In many old persons, also, labouring under enlargement of the prostate gland, the difficulties attendant on the introduction of the instrument must be greatly increased, and the success of the operation less probable, from the greater likelihood of retention of some of the fragments of the calculus. At the same time, the attempts could never occasion the danger and constitutional disturbance consequent on the operation of lithotomy.

Again, this operation would be more likely to succeed in those cases where the stone, being composed chiefly of the triple phosphate, is soft and friable, than where the calculus, formed principally of lithic acid, is hard and compact; and the division of such a calculus into several irregular fragments might occasion more inconvenience than the presence of the entire calculus in the bladder.

The practice of detecting disease of the thoracic cavity by means of auscultation, is another improvement, for which we are indebted to the French: as yet it has not become general in England. In order to arrive at a correct diagnosis by means of the stethoscope, a long period of study and practice is requisite; and from the diagnostic errors into which it led practitioners, who relied too exclusively upon it, and who, perhaps, were not sufficiently accustomed to its use, it is not in such general use on the continent as formerly.

The practice of *torsion*, or twisting the extremities of arteries after operations, in order to supersede the necessity of ligatures, was for a short period partially followed by a few French surgeons: this plan presented so little advantage, in comparison to the risk from hemorrhage, over the simple and efficacious method of the ligature, that it was never likely to be generally adopted.

Having thus taken a cursory glance at some of the principal points of difference in practice between England and France, although I cannot come to the conclusion at which

M. Roux arrives, that French practice is more generally good than English, yet many points of French practice might be adopted with advantage in England: as, on the other hand, the treatment of disease in France might, in many instances, be improved, by their practitioners availing themselves of some parts of the practice peculiar to this country.

(To be continued.) 381.

---

### HOSPITAL REPORTS.

